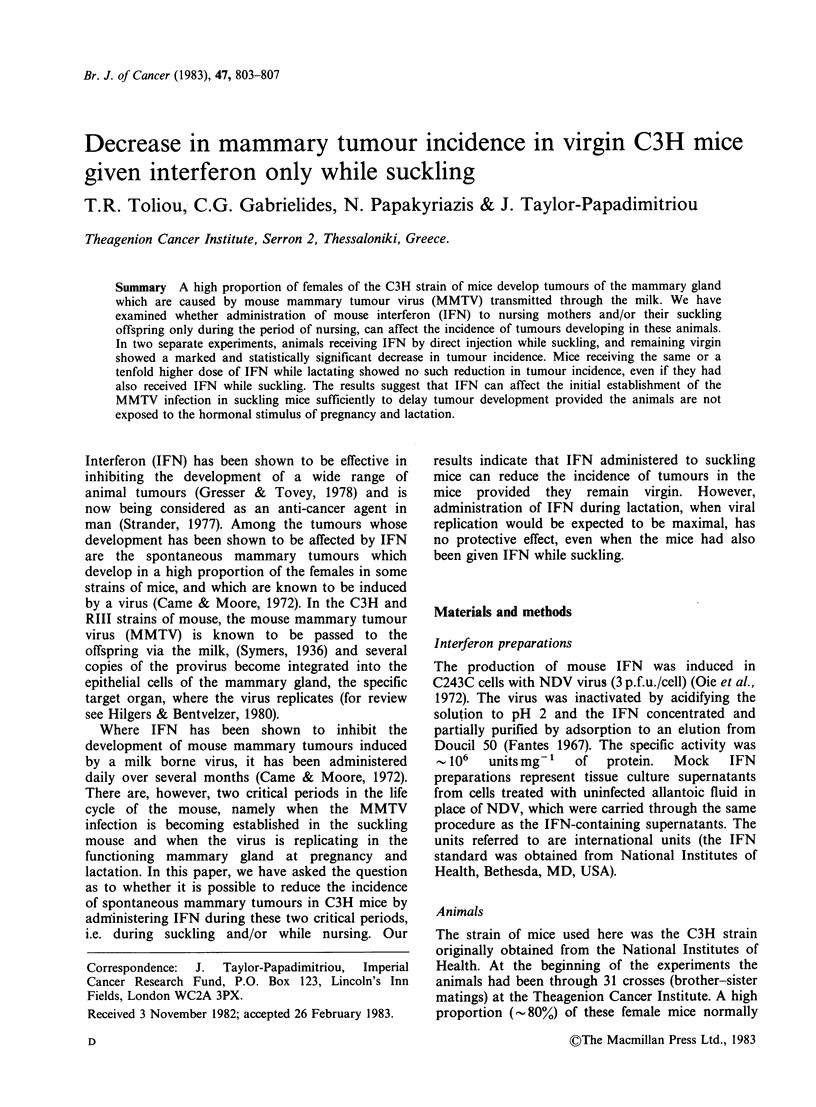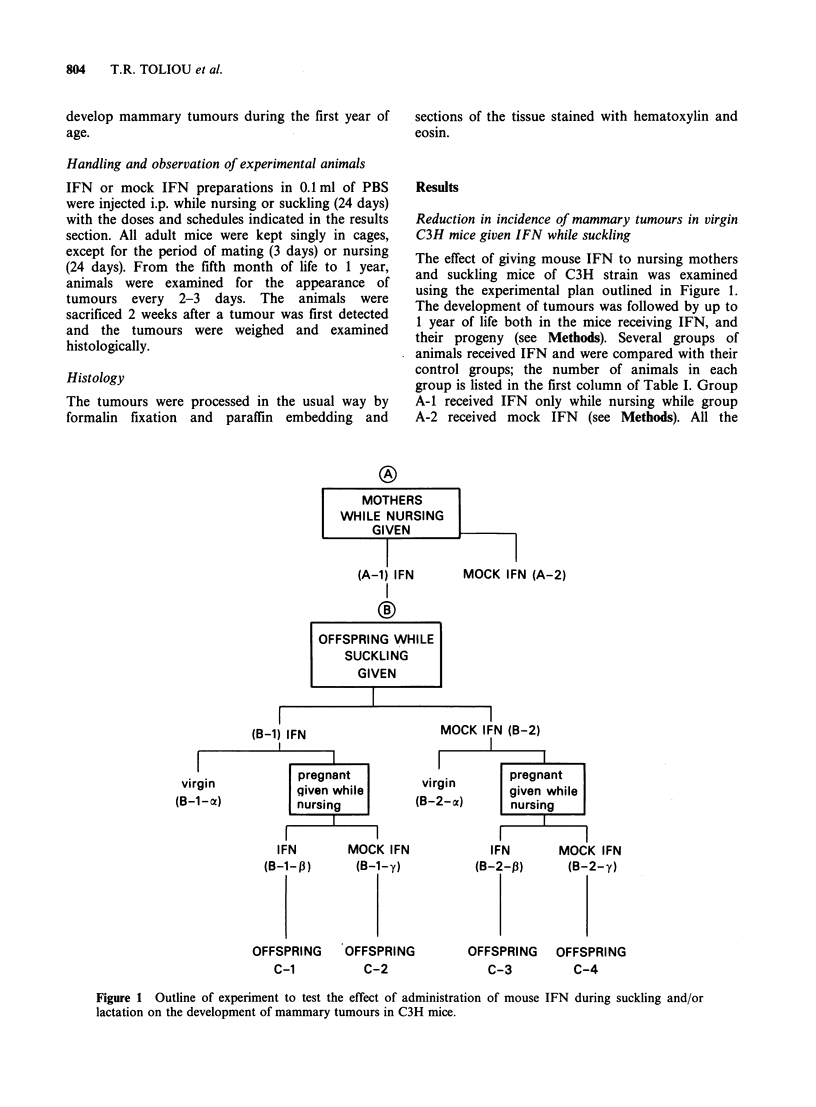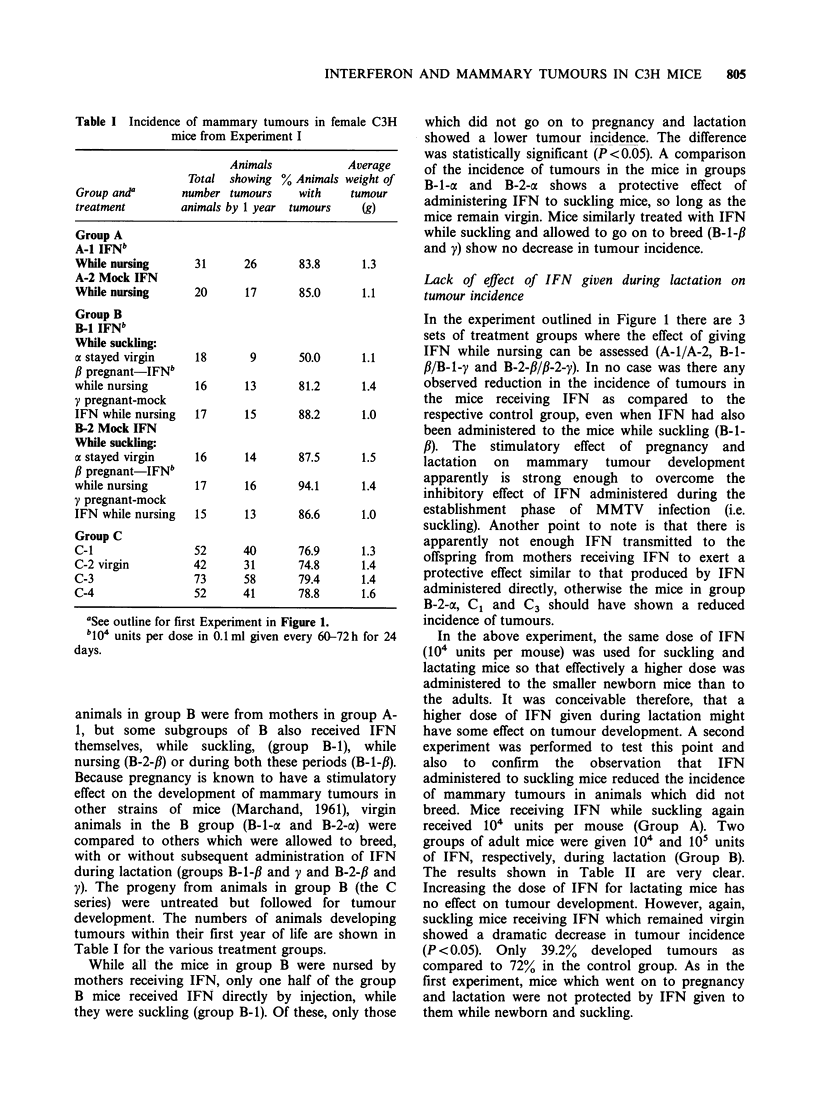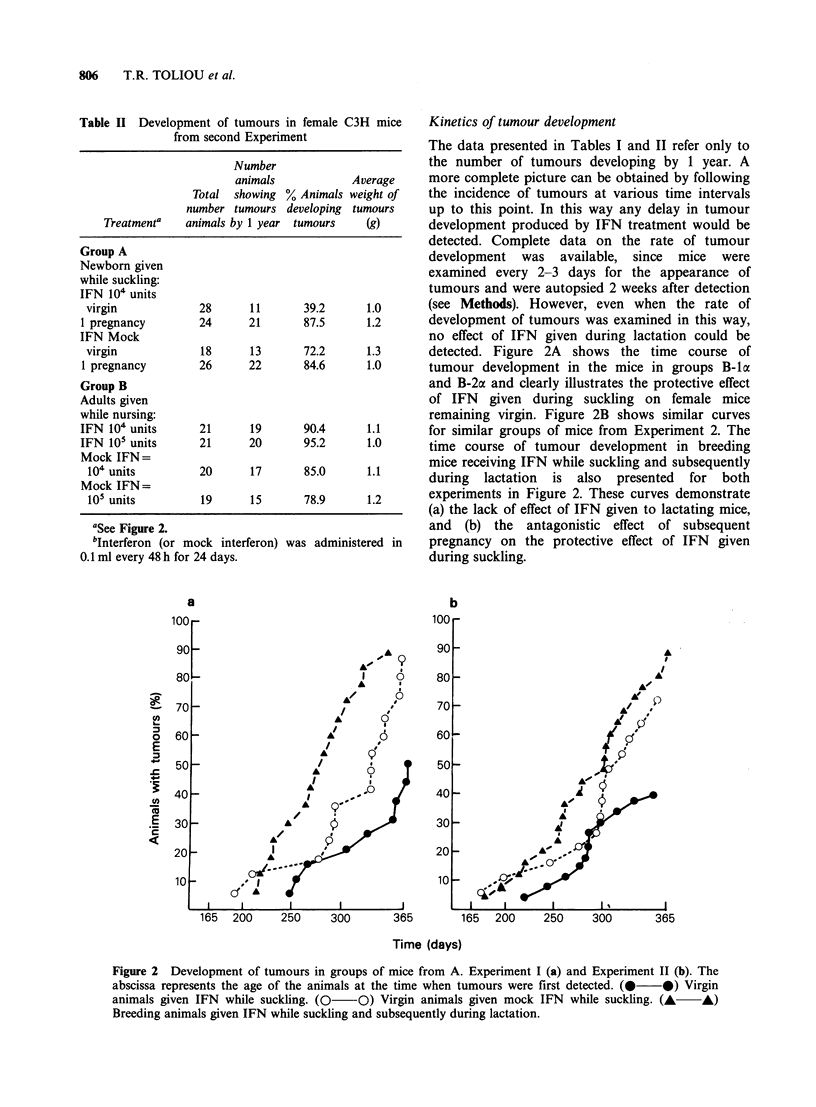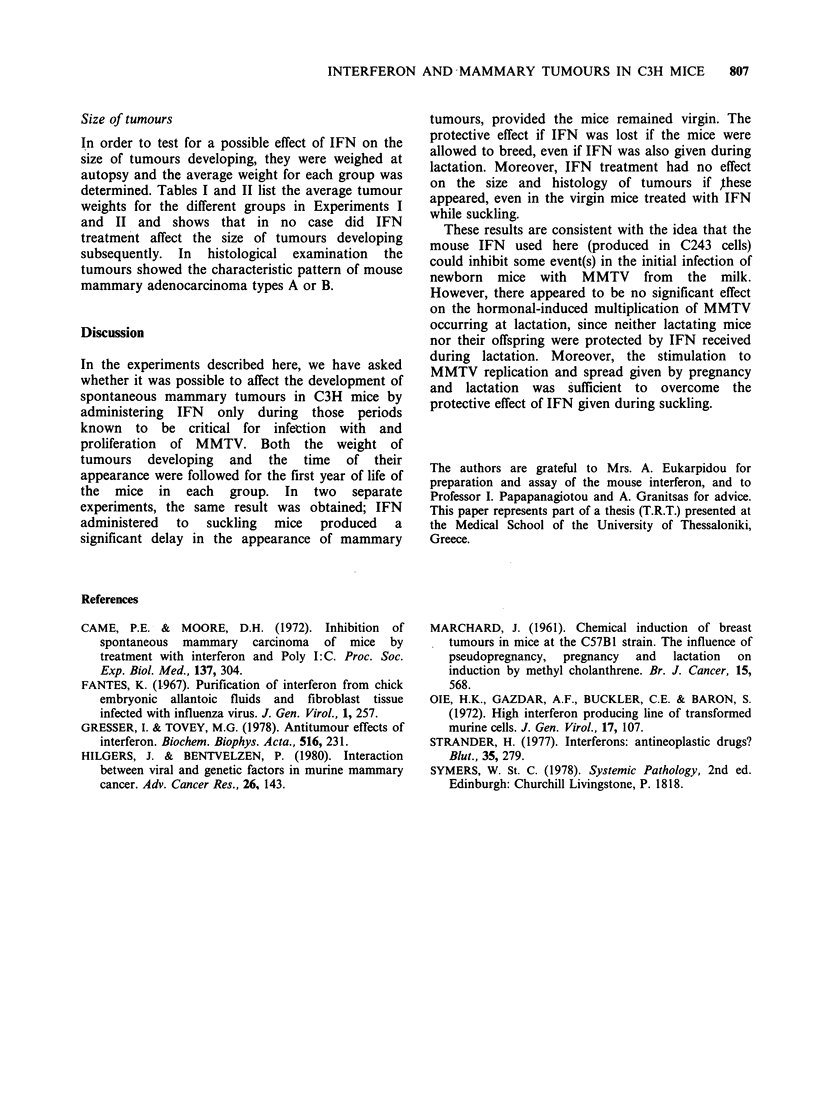# Decrease in mammary tumour incidence in virgin C3H mice given interferon only while suckling.

**DOI:** 10.1038/bjc.1983.134

**Published:** 1983-06

**Authors:** T. R. Toliou, C. G. Gabrielides, N. Papakyriazis, J. Taylor-Papadimitriou

## Abstract

A high proportion of females of the C3H strain of mice develop tumours of the mammary gland which are caused by mouse mammary tumour virus (MMTV) transmitted through the milk. We have examined whether administration of mouse interferon (IFN) to nursing mothers and/or their suckling offspring only during the period of nursing, can affect the incidence of tumours developing in these animals. In two separate experiments, animals receiving IFN by direct injection while suckling, and remaining virgin showed a marked and statistically significant decrease in tumour incidence. Mice receiving the same or a tenfold higher dose of IFN while lactating showed no such reduction in tumour incidence, even if they had also received IFN while suckling. The results suggest that IFN can affect the initial establishment of the MMTV infection in suckling mice sufficiently to delay tumour development provided the animals are not exposed to the hormonal stimulus of pregnancy and lactation.


*Br. J. of Cancer* (1983), **47**, 803–807

### Decrease in mammary tumour incidence in virgin C3H mice given interferon only while suckling

T.R. Toliou, C.G. Gabrielides, N. Papakyriazis & J. Taylor-Papadimitriou

*Theagenion Cancer Institute, Serron 2, Thessaloniki, Greece.*

**Summary** A high proportion of females of the C3H strain of mice develop tumours of the mammary gland which are caused by mouse mammary tumour virus (MMTV) transmitted through the milk. We have examined whether administration of mouse interferon (IFN) to nursing mothers and/or their suckling offspring only during the period of nursing, can affect the incidence of tumours developing in these animals. In two separate experiments, animals receiving IFN by direct injection while suckling, and remaining virgin showed a marked and statistically significant decrease in tumour incidence. Mice receiving the same or a tenfold higher dose of IFN while lactating showed no such reduction in tumour incidence, even if they had also received IFN while suckling. The results suggest that IFN can affect the initial establishment of the MMTV infection in suckling mice sufficiently to delay tumour development provided the animals are not exposed to the hormonal stimulus of pregnancy and lactation.

Interferon (IFN) has been shown to be effective in inhibiting the development of a wide range of animal tumours (Gresser & Tovey, 1978) and is now being considered as an anti-cancer agent in man (Strander, 1977). Among the tumours whose development has been shown to be affected by IFN are the spontaneous mammary tumours which develop in a high proportion of the females in some strains of mice, and which are known to be induced by a virus (Came & Moore, 1972). In the C3H and RIII strains of mouse, the mouse mammary tumour virus (MMTV) is known to be passed to the offspring via the milk, (Symers, 1936) and several copies of the provirus become integrated into the epithelial cells of the mammary gland, the specific target organ, where the virus replicates (for review see Hilgers & Bentvelzer, 1980).

Where IFN has been shown to inhibit the development of mouse mammary tumours induced by a milk borne virus, it has been administered daily over several months (Came & Moore, 1972). There are, however, two critical periods in the life cycle of the mouse, namely when the MMTV infection is becoming established in the suckling mouse and when the virus is replicating in the functioning mammary gland at pregnancy and lactation. In this paper, we have asked the question as to whether it is possible to reduce the incidence of spontaneous mammary tumours in C3H mice by administering IFN during these two critical periods, i.e. during suckling and/or while nursing. Our results indicate that IFN administered to suckling mice can reduce the incidence of tumours in the mice provided they remain virgin. However, administration of IFN during lactation, when viral replication would be expected to be maximal, has no protective effect, even when the mice had also been given IFN while suckling.

Correspondence: J. Taylor-Papadimitriou, Imperial Cancer Research Fund, P.O. Box 123, Lincoln's Inn Fields, London WC2A 3PX.

Received 3 November 1982; accepted 26 February 1983.

### Materials and methods

### *Interferon preparations*

The production of mouse IFN was induced in C243C cells with NDV virus (3 p.f.u./cell) (Oie *et al.*, 1972). The virus was inactivated by acidifying the solution to pH 2 and the IFN concentrated and partially purified by adsorption to an elution from Doucil 50 (Fantes 1967). The specific activity was $\sim 10^6$ units $mg^{-1}$ of protein. Mock IFN preparations represent tissue culture supernatants from cells treated with uninfected allantoic fluid in place of NDV, which were carried through the same procedure as the IFN-containing supernatants. The units referred to are international units (the IFN standard was obtained from National Institutes of Health, Bethesda, MD, USA).

### *Animals*

The strain of mice used here was the C3H strain originally obtained from the National Institutes of Health. At the beginning of the experiments the animals had been through 31 crosses (brother–sister matings) at the Theagenion Cancer Institute. A high proportion ($\sim$80%) of these female mice normally

©The Macmillan Press Ltd., 1983

develop mammary tumours during the first year of age.

*Handling and observation of experimental animals*

IFN or mock IFN preparations in 0.1 ml of PBS were injected i.p. while nursing or suckling (24 days) with the doses and schedules indicated in the results section. All adult mice were kept singly in cages, except for the period of mating (3 days) or nursing (24 days). From the fifth month of life to 1 year, animals were examined for the appearance of tumours every 2–3 days. The animals were sacrificed 2 weeks after a tumour was first detected and the tumours were weighed and examined histologically.

*Histology*

The tumours were processed in the usual way by formalin fixation and paraffin embedding and sections of the tissue stained with hematoxylin and eosin.

### Results

*Reduction in incidence of mammary tumours in virgin C3H mice given IFN while suckling*

The effect of giving mouse IFN to nursing mothers and suckling mice of C3H strain was examined using the experimental plan outlined in Figure 1. The development of tumours was followed by up to 1 year of life both in the mice receiving IFN, and their progeny (see **Methods**). Several groups of animals received IFN and were compared with their control groups; the number of animals in each group is listed in the first column of Table I. Group A-1 received IFN only while nursing while group A-2 received mock IFN (see **Methods**). All the

**Figure 1** Outline of experiment to test the effect of administration of mouse IFN during suckling and/or lactation on the development of mammary tumours in C3H mice.

**Table I** Incidence of mammary tumours in female C3H mice from Experiment I

| *Group and*[a] *treatment* | *Total number animals* | *Animals showing tumours by 1 year* | *% Animals with tumours* | *Average weight of tumour (g)* |
|---|---|---|---|---|
| **Group A** | | | | |
| **A-1 IFN**[b] | | | | |
| **While nursing** | 31 | 26 | 83.8 | 1.3 |
| **A-2 Mock IFN** | | | | |
| **While nursing** | 20 | 17 | 85.0 | 1.1 |
| **Group B** | | | | |
| **B-1 IFN**[b] | | | | |
| **While suckling:** | | | | |
| α stayed virgin | 18 | 9 | 50.0 | 1.1 |
| β pregnant—IFN[b] while nursing | 16 | 13 | 81.2 | 1.4 |
| γ pregnant-mock IFN while nursing | 17 | 15 | 88.2 | 1.0 |
| **B-2 Mock IFN** | | | | |
| **While suckling:** | | | | |
| α stayed virgin | 16 | 14 | 87.5 | 1.5 |
| β pregnant—IFN[b] while nursing | 17 | 16 | 94.1 | 1.4 |
| γ pregnant-mock IFN while nursing | 15 | 13 | 86.6 | 1.0 |
| **Group C** | | | | |
| C-1 | 52 | 40 | 76.9 | 1.3 |
| C-2 virgin | 42 | 31 | 74.8 | 1.4 |
| C-3 | 73 | 58 | 79.4 | 1.4 |
| C-4 | 52 | 41 | 78.8 | 1.6 |

[a]See outline for first Experiment in **Figure 1.**
[b]$10^4$ units per dose in 0.1 ml given every 60–72 h for 24 days.

animals in group B were from mothers in group A-1, but some subgroups of B also received IFN themselves, while suckling, (group B-1), while nursing (B-2-β) or during both these periods (B-1-β). Because pregnancy is known to have a stimulatory effect on the development of mammary tumours in other strains of mice (Marchand, 1961), virgin animals in the B group (B-1-α and B-2-α) were compared to others which were allowed to breed, with or without subsequent administration of IFN during lactation (groups B-1-β and γ and B-2-β and γ). The progeny from animals in group B (the C series) were untreated but followed for tumour development. The numbers of animals developing tumours within their first year of life are shown in Table I for the various treatment groups.

While all the mice in group B were nursed by mothers receiving IFN, only one half of the group B mice received IFN directly by injection, while they were suckling (group B-1). Of these, only those which did not go on to pregnancy and lactation showed a lower tumour incidence. The difference was statistically significant ($P<0.05$). A comparison of the incidence of tumours in the mice in groups B-1-α and B-2-α shows a protective effect of administering IFN to suckling mice, so long as the mice remain virgin. Mice similarly treated with IFN while suckling and allowed to go on to breed (B-1-β and γ) show no decrease in tumour incidence.

*Lack of effect of IFN given during lactation on tumour incidence*

In the experiment outlined in Figure 1 there are 3 sets of treatment groups where the effect of giving IFN while nursing can be assessed (A-1/A-2, B-1-β/B-1-γ and B-2-β/β-2-γ). In no case was there any observed reduction in the incidence of tumours in the mice receiving IFN as compared to the respective control group, even when IFN had also been administered to the mice while suckling (B-1-β). The stimulatory effect of pregnancy and lactation on mammary tumour development apparently is strong enough to overcome the inhibitory effect of IFN administered during the establishment phase of MMTV infection (i.e. suckling). Another point to note is that there is apparently not enough IFN transmitted to the offspring from mothers receiving IFN to exert a protective effect similar to that produced by IFN administered directly, otherwise the mice in group B-2-α, $C_1$ and $C_3$ should have shown a reduced incidence of tumours.

In the above experiment, the same dose of IFN ($10^4$ units per mouse) was used for suckling and lactating mice so that effectively a higher dose was administered to the smaller newborn mice than to the adults. It was conceivable therefore, that a higher dose of IFN given during lactation might have some effect on tumour development. A second experiment was performed to test this point and also to confirm the observation that IFN administered to suckling mice reduced the incidence of mammary tumours in animals which did not breed. Mice receiving IFN while suckling again received $10^4$ units per mouse (Group A). Two groups of adult mice were given $10^4$ and $10^5$ units of IFN, respectively, during lactation (Group B). The results shown in Table II are very clear. Increasing the dose of IFN for lactating mice has no effect on tumour development. However, again, suckling mice receiving IFN which remained virgin showed a dramatic decrease in tumour incidence ($P<0.05$). Only 39.2% developed tumours as compared to 72% in the control group. As in the first experiment, mice which went on to pregnancy and lactation were not protected by IFN given to them while newborn and suckling.

**Table II** Development of tumours in female C3H mice from second Experiment

| *Treatment*[a] | *Total number animals* | *Number animals showing tumours by 1 year* | *% Animals developing tumours* | *Average weight of tumours (g)* |
|---|---|---|---|---|
| **Group A** | | | | |
| Newborn given while suckling: | | | | |
| IFN $10^4$ units | | | | |
| virgin | 28 | 11 | 39.2 | 1.0 |
| 1 pregnancy | 24 | 21 | 87.5 | 1.2 |
| IFN Mock | | | | |
| virgin | 18 | 13 | 72.2 | 1.3 |
| 1 pregnancy | 26 | 22 | 84.6 | 1.0 |
| **Group B** | | | | |
| Adults given while nursing: | | | | |
| IFN $10^4$ units | 21 | 19 | 90.4 | 1.1 |
| IFN $10^5$ units | 21 | 20 | 95.2 | 1.0 |
| Mock IFN = $10^4$ units | 20 | 17 | 85.0 | 1.1 |
| Mock IFN = $10^5$ units | 19 | 15 | 78.9 | 1.2 |

[a]See **Figure 2.**
[b]Interferon (or mock interferon) was administered in 0.1 ml every 48 h for 24 days.

*Kinetics of tumour development*

The data presented in Tables I and II refer only to the number of tumours developing by 1 year. A more complete picture can be obtained by following the incidence of tumours at various time intervals up to this point. In this way any delay in tumour development produced by IFN treatment would be detected. Complete data on the rate of tumour development was available, since mice were examined every 2–3 days for the appearance of tumours and were autopsied 2 weeks after detection (see **Methods**). However, even when the rate of development of tumours was examined in this way, no effect of IFN given during lactation could be detected. Figure 2A shows the time course of tumour development in the mice in groups B-1α and B-2α and clearly illustrates the protective effect of IFN given during suckling on female mice remaining virgin. Figure 2B shows similar curves for similar groups of mice from Experiment 2. The time course of tumour development in breeding mice receiving IFN while suckling and subsequently during lactation is also presented for both experiments in Figure 2. These curves demonstrate (a) the lack of effect of IFN given to lactating mice, and (b) the antagonistic effect of subsequent pregnancy on the protective effect of IFN given during suckling.

**Figure 2** Development of tumours in groups of mice from A. Experiment I (**a**) and Experiment II (**b**). The abscissa represents the age of the animals at the time when tumours were first detected. (●——●) Virgin animals given IFN while suckling. (○——○) Virgin animals given mock IFN while suckling. (▲——▲) Breeding animals given IFN while suckling and subsequently during lactation.

*Size of tumours*

In order to test for a possible effect of IFN on the size of tumours developing, they were weighed at autopsy and the average weight for each group was determined. Tables I and II list the average tumour weights for the different groups in Experiments I and II and shows that in no case did IFN treatment affect the size of tumours developing subsequently. In histological examination the tumours showed the characteristic pattern of mouse mammary adenocarcinoma types A or B.

### Discussion

In the experiments described here, we have asked whether it was possible to affect the development of spontaneous mammary tumours in C3H mice by administering IFN only during those periods known to be critical for infection with and proliferation of MMTV. Both the weight of tumours developing and the time of their appearance were followed for the first year of life of the mice in each group. In two separate experiments, the same result was obtained; IFN administered to suckling mice produced a significant delay in the appearance of mammary tumours, provided the mice remained virgin. The protective effect if IFN was lost if the mice were allowed to breed, even if IFN was also given during lactation. Moreover, IFN treatment had no effect on the size and histology of tumours if these appeared, even in the virgin mice treated with IFN while suckling.

These results are consistent with the idea that the mouse IFN used here (produced in C243 cells) could inhibit some event(s) in the initial infection of newborn mice with MMTV from the milk. However, there appeared to be no significant effect on the hormonal-induced multiplication of MMTV occurring at lactation, since neither lactating mice nor their offspring were protected by IFN received during lactation. Moreover, the stimulation to MMTV replication and spread given by pregnancy and lactation was sufficient to overcome the protective effect of IFN given during suckling.

The authors are grateful to Mrs. A. Eukarpidou for preparation and assay of the mouse interferon, and to Professor I. Papapanagiotou and A. Granitsas for advice. This paper represents part of a thesis (T.R.T.) presented at the Medical School of the University of Thessaloniki, Greece.

### References

CAME, P.E. & MOORE, D.H. (1972). Inhibition of spontaneous mammary carcinoma of mice by treatment with interferon and Poly I:C. *Proc. Soc. Exp. Biol. Med.*, **137**, 304.

FANTES, K. (1967). Purification of interferon from chick embryonic allantoic fluids and fibroblast tissue infected with influenza virus. *J. Gen. Virol.*, **1**, 257.

GRESSER, I. & TOVEY, M.G. (1978). Antitumour effects of interferon. *Biochem. Biophys. Acta.*, **516**, 231.

HILGERS, J. & BENTVELZEN, P. (1980). Interaction between viral and genetic factors in murine mammary cancer. *Adv. Cancer Res.*, **26**, 143.

MARCHARD, J. (1961). Chemical induction of breast tumours in mice at the C57B1 strain. The influence of pseudopregnancy, pregnancy and lactation on induction by methyl cholanthrene. *Br. J. Cancer*, **15**, 568.

OIE, H.K., GAZDAR, A.F., BUCKLER, C.E. & BARON, S. (1972). High interferon producing line of transformed murine cells. *J. Gen. Virol.*, **17**, 107.

STRANDER, H. (1977). Interferons: antineoplastic drugs? *Blut.*, **35**, 279.

SYMERS, W. St. C. (1978). *Systemic Pathology*, 2nd ed. Edinburgh: Churchill Livingstone, P. 1818.